# Prevalence of recent occupational injury and its associated factors among food industry workers in Selangor

**DOI:** 10.1371/journal.pone.0293987

**Published:** 2023-11-09

**Authors:** Rahmat Dapari, Mohd Hafizuddin Mahfot, Florence Chiu Yan Yee, Aisyah Nabilah Iftikhar Ahmad, Krishen Magayndran, Mohd ‘Ammar Ihsan Ahmad Zamzuri, Zaleha Md Isa, Mohd Rohaizat Hassan, Nazri Che Dom, Syed Sharizman Syed Abdul Rahim

**Affiliations:** 1 Department of Community Health, Universiti Putra Malaysia, Serdang, Malaysia; 2 State Department of Health Negeri Sembilan, Seremban, Negeri Sembilan, Malaysia; 3 Department of Community Health, Universiti Kebangsaan Malaysia, Cheras, Malaysia; 4 Faculty of Health Sciences, Universiti Teknologi MARA, Bandar Puncak Alam, Malaysia; 5 Public Health Medicine Department, Faculty of Medicine and Health Sciences, Universiti Malaysia Sabah, Kota Kinabalu, Malaysia; Wroclaw University of Environmental and Life Sciences: Uniwersytet Przyrodniczy we Wroclawiu, POLAND

## Abstract

**Introduction:**

Malaysia’s gross domestic product is heavily influenced by the food and beverage sector and the contribution of the industry to the national economy is expected to increase in the coming years. Thus, the need for employees in the food industry will continue to rise as this sector grows. Nevertheless, employees in the food industry are exposed to various occupational hazards that can lead to occupational injuries, mainly related to kitchen work. Given the increasing number of employees in the food industry and the rising trend of occupational injuries, this study was conducted to determine the prevalence of recent occupational injuries and their associated factors and predictors among food industry workers.

**Methods:**

This cross-sectional study was conducted in 2023 among food industry workers in Selangor, Malaysia. The respondents were sampled using a multistage random sampling method. Data were collected via online self-administered questionnaires and analysed using descriptive statistics and logistic regression models in the SPSS software, version 25.

**Results:**

A total of 250 responses were received from 342 samples, with an overall response rate of 73.0%. The prevalence of recent occupational injuries among food industry workers was 44.8%. Statistically, significant associations were present between occupational injuries and alcohol consumption (p = 0.001), poor knowledge (p = 0.031), poor compliance (p = 0.021), poor safety management (p = 0.021), poor safety training (p = 0.002), poor safety culture (p = 0.003), physical exposure (p < 0.001), and ergonomic exposure (p = 0.009). The predictors for recent occupational injuries among food industry workers were Malay (adjusted Odds Ratio; aOR = 2.60, p = 0.027, 95% Confidence Interval; CI = 1.116, 6.035), alcohol consumption (aOR = 5.31, p = 0.001, 95% CI = 2.042, 13.779), poor knowledge (aOR = 1.98, p = 0.032, 95% CI = 1.059, 3.691), poor safety culture (aOR = 2.44, p = 0.002, 95% CI = 1.372, 4.342), and exposure to physical hazards (aOR = 8.88, p < 0.001, 95% CI = 3.031, 26.014).

**Conclusion:**

This study has found a high prevalence of occupational injuries among food industry workers, thereby highlighting the importance of addressing alcohol consumption, improving worker knowledge, enhancing work safety culture, and better control measures on exposure to physical hazards, especially among Malay workers. By prioritising these factors, employers can create safer work environments and minimise the risk of occupational injuries.

## Introduction

Food industry workers are defined as individuals who are directly involved in food preparation, come into contact with food or food contact surfaces, and handle packaged or unpackaged food, or appliances on any food premises [1–3]. The importance of the food industry can be viewed from three aspects, namely income generation, Gross Domestic Product (GDP), and employment. The food industry is the largest industrial manufacturing sector in most nations in the European Union, contributing to annual sales of more than €1.109 trillion and employing more than 4.57 million people [4, 5]. In Malaysia, the GDP is heavily influenced by the food industry and this contribution is anticipated to increase in the coming years [6]. Thus, the demand for employees in the food industry will continue to rise as this sector grows. Nonetheless, employees in the food industry are exposed to diverse occupational hazards that may culminate in occupational injuries [7–11].

An occupational injury is defined as any personal injury, disease or death resulting from an occupational accident [12]. An occupational accident is an unexpected and unplanned occurrence, including acts of violence, arising out of or in connection with work, which results in one or more workers incurring a personal injury, disease or death [12]. Occupational hazards in the global food industry, including restaurants and beverage stores, constitute considerable risks to injury among workers. Slips, trips, falls, burns, cuts, and musculoskeletal difficulties are frequently impacted by factors such as poor training, occupational stress, exhaustion, equipment and infrastructure concerns, and a lack of suitable personal protection equipment (PPE) [6–8]. Understanding these injuries and their causes is crucial as it promotes employee safety culture, ensures legal compliance, boosts productivity and efficiency, fosters a positive industry reputation, and contributes to the long-term sustainability of the food industry, resulting in a safer and more prosperous working environment for all involved [5, 13].

According to the International Labour Organization (ILO), it is estimated that 2.78 million occupational fatalities and 374 million non-fatal occupational injuries and illnesses occur globally each year [14]. Manufacturing industries, including the food and beverage sector, accounted for 14.1% of nonfatal occupational injuries and illnesses in private industries in 2020, while 7.2% were from food services [14]. A study in Greece found that 44.3% of restaurant workers have experienced at least one occupational accident and 56.7% have no idea about the dangers they were exposed to at their workplace [15]. The exposure to occupational hazards, particularly in lower and middle-income countries, is higher given the limited safety resources and wide range of hazards including chemical, biological, psychosocial and ergonomic hazards [3]. Factors such as scarcity of resources, lack of well-equipped facilities, personnel shortages, overcrowding, lack of training, and improper execution of safety guidelines and regulations are key factors increasing the risk of occupational accidents [6–8, 15]. These diverse factors underscore the importance of adequate and effective implementation of occupational safety policies and regulations.

The occupational safety and health of food industry workers in Malaysia are generally governed and regulated by the Occupational Safety and Health (OSHA) Act 1994 (Act 514), similar to any other occupation [13]. In terms of occupational injuries categorised by sectors in Malaysia, an increasing trend of accidents was observed among food industry workers annually. In 2020, 62% of accidents with temporary disability, 84% of accidents with a permanent disability, and 34% of deaths reported to DOSH came from the manufacturing industries, including the food industry [16]. Occupational injuries among food industry workers are often related to kitchen work where workers could suffer a variety of injuries, such as falls, burns, cuts, strains, and eye problems [17].

Selangor’s food industry thrives, with a varied range of sizes including roadside stalls, restaurants, homemade meals, food riders, frozen food and pastry manufacturers. This wide range of food services requires the handling of hazardous materials and constant movement over short and long distances, thereby increasing the exposure of food industry workers to occupational hazards and the risk of occupational injuries. Occupational injuries in the food industry not only result in personal suffering for workers but also lead to increased healthcare costs and reduced productivity. This study focuses on establishing the prevalence of recent occupational injuries, as well as their associated factors and predictors among food industry workers. Understanding these issues is critical for improving worker safety, lowering economic burdens, ensuring legal compliance, mitigating public health risks, informing effective policies, and promoting long-term industry sustainability. The research findings can be used by policymakers and stakeholders to develop effective intervention programmes in reducing injury among high-risk groups of food industry workers.

## Methods

### Study design and study location

**Fig 1:**
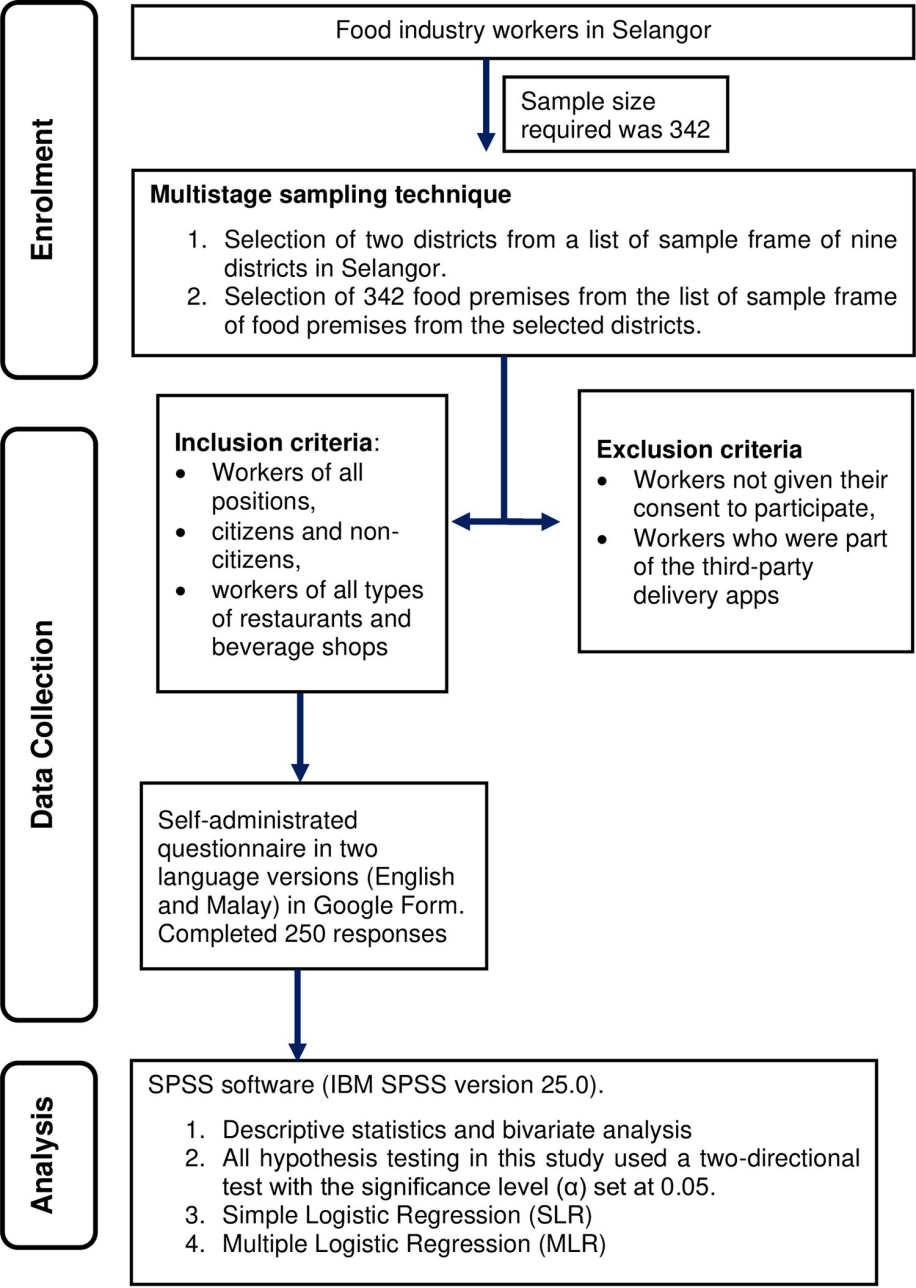
Schematic diagram of study design.

A cross-sectional study was conducted among food industry workers in Selangor. The total population in Selangor is currently estimated at 6.54 million citizens and 0.5 million non-citizens, with 3.71 million males and 3.32 million females [18]. Fig 1 displays the schematic diagram of the study design. The inclusion criteria for this study were as follows: i) food industry workers of all positions, including in-house delivery and dispatch workers, ii) citizens and non-citizens, and iii) workers of all types of restaurants and beverage shops located in Selangor. The exclusion criteria were as follows: i) food industry workers who have not given their consent to participate, and ii) workers who were part of third-party delivery apps, such as Grab and Foodpanda.

### Sampling frame and sample size

The required sample size for each potentially associated factor was estimated and the highest estimated number was used. The sample size for this study was calculated using the two-proportions formula. Accordingly, the required sample size for this study to obtain 95% precision and 80% power was estimated to be 285 based on the investigated factors associated with occupational injuries [19]. The final sample size was calculated as 342 upon adjusting for a 20% dropout rate.

### Study instruments and data collection

A self-administered questionnaire in two language versions (English and Malay) in Google Form was used in this study. The questionnaire was divided into three sections. Section 1 consisted of 13 mixed questions that were designed to collect relevant data on the respondents’ sociodemographic, socioeconomic, and lifestyle factors. Section 2 comprised 1 question each on the recent and type of occupational injury. Section 3 consisted of 12 subsections on latent and observed variables of occupational factors. The latent variables comprised knowledge, compliance, safety management, safety training, and safety culture, whereas the observed variables of occupational factors included physical exposure, chemical exposure, ergonomic exposure, rest at work, working status, working hours, and years of working experience. The Cronbach alpha values for the latent variables ranged from 0.70 to 0.87, which are all considered acceptable.

A multistage sampling technique was used for data collection. The first stage of sampling was the selection of two districts from a list of sample frames of nine districts in Selangor. The second stage of sampling was the selection of 342 food premises from the list of sample frames of food premises from the selected districts.

The respondents were assessed for eligibility before being invited to participate in this study. This method was selected to obtain a workable sample size. Data collection was performed gradually from May to June 2023 until the required sample size was obtained. The respondents were approached physically at food and beverage outlets. The objective of this study was explained to each outlet manager and permission to approach the employees was obtained.

### Data analysis

The collected data from the questionnaires were analysed using SPSS software (IBM SPSS version 25.0). Numerical data were assessed for normality using descriptive statistics and presented as median and interquartile range because they were not normally distributed. For the categorical data, frequency and percentage were used to describe the respondents’ characteristics. A two-directional test was conducted for all the hypotheses with the significance level (α) at 0.05. The Chi-square and Fisher’s Exact Tests were used to determine the association between the prevalence of occupational injuries and all the categorical independent variables. The associations among variables with numerical data were examined using a non-parametric test, as they were not normally distributed. All potential variables were subsequently tested using Simple Logistic Regression (SLR) and Multiple Logistic Regression (MLR) to determine the significant predictors and adjusted odds ratio (aOR) at 95% Confidence Intervals (CI).

### Ethics statement

Ethical approval was obtained from the Ethics Committee for Research Involving Human Subjects of Universiti Putra Malaysia (JKEUPM– 2023–061) on 3^rd^ March 2023. Consent was obtained from each participant using an online consent form, following their verbal agreement to participate in this study before administering the questionnaire. No minors were involved in this study and participants were allowed to withdraw at any point without any penalty.

## Results

A total of 250 responses were received from 342 distributed samples, with an overall response rate of 73.0%. The three numerical variables, namely age, number of working years, and monthly salary were not normally distributed.

Table 1 depicts that the prevalence of recent occupational injuries among food industry workers is at 44.8%. The majority of food industry workers who experienced recent occupational injury have reported getting cut (23.2%), followed by fall (15.2%), burn (14.3%), lower back pain (10.7%), and sprain (7.1%), as shown in Table 2.

**Table 1:** Prevalence of recent occupational injury.

Variable	Recent occupational injury (1 year)		Total number of food industry workers
	Yes (%)	No (%)	
Number of food industry workers	112 (44.8%)	138 (55.2%)	250

**Table 2:** Types of recent occupational injury (n = 112).

No.	Types of injury	Number of workers (%)
1.	Cut	26 (23.2%)
2.	Fall	17 (15.2%)
3.	Burn	16 (14.3%)
4.	Lower Back Pain	12 (10.7%)
5.	Sprain	8 (7.1%)
6.	Burn, Cut	6 (5.4%)
7.	Cut, Fall, Burn, Sprain	4 (3.6%)
8.	Fall, Cut	3 (2.7%)
9.	Joint pain	3 (2.7%)
10.	Not specified	17 (15.2%)

Descriptively, the respondents’ median age is 26 (11%), with the majority being male (57.2%), Malay (56.4%), a citizen (90.8%), and having attained secondary education (49.6%) (Table 2). Regarding their health behaviour, 32% of the respondents were smokers and 5.6% reported they would smoke at work; 22% of them vape and 6.4% reported they would vape at work. Meanwhile, 14.4% of the respondents reported that they consume alcohol and 3.6% reported that they would drink at work.

As shown in Table 3, most respondents have poor knowledge (70.8%), poor compliance (73.2%), good management (63.2%), good training (60.8%), and good work safety culture (59.2%). In addition, a higher proportion of the respondents are exposed to physical (85.2%), chemical (64.0%), and ergonomic hazards (79.2%). More than 60.0% of the respondents claimed to have adequate rest (70.8%), were permanent workers (74.0%), and worked long working hours of more than 45 hours per week (63.6%). A sub-analysis of two items under the work safety culture revealed that 37.2% and 42.0% of respondents agreed and strongly agreed that safety training was provided to all workers in the food industry. Nevertheless, a substantial percentage of respondents agreed (23.6%) and strongly agreed (24.0%) to pay for the cost of safety training, if it is offered.

**Table 3:** Association between recent occupational injury and characteristics of respondents in Selangor.

Variable	n (%)	Recent Occupational Injury		P value
		Yes	No	
		112 (44.8%)	138 (55.2%)	
**Age** ^**a**^	26 (11)^#^	26 (10)^#^	27 (12)^#^	0.519*
**Gender**				
Female	107 (42.8)	49 (45.8)	58(54.2)	0.784
Male	143 (57.2)	63 (44.1)	80(55.9)	
**Ethnicity**				
Malay	141 (56.4)	65 (46.1)	76 (53.9)	0.575
Chinese	49 (19.6)	20 (40.8)	29 (59.2)	
Indian	37 (14.8)	19 (51.4)	18 (48.6)	
Others	23 (9.2)	8 (34.8)	15 (65.2)	
**Nationality**				
Citizen	227 (90.8)	104 (45.8)	123 (54.2)	0.311
Non-citizen	23 (9.2)	8 (34.8)	15 (65.2)	
**Educational level**				
Primary	21 (8.4)	14 (66.7)	7 (33.3)	0.290
Secondary	124 (49.6)	55 (44.4)	69 (55.6)	
Diploma	74 (29.6)	33 (44.6)	41 (55.4)	
Bachelor	11 (4.4)	4 (36.4)	7 (63.6)	
Master	7 (2.8)	2 (28.6)	5 (71.4)	
Others	13 (5.2)	4 (30.8)	9 (69.2)	
**Smoking status**				
Smoking	80 (32.0)	40 (50.0)	40 (50.0)	0.257
Not smoking	170 (68.0)	72 (42.4)	98 (57.6)	
**Smoking during work**				
Smoking during work	14 (5.6)	9 (64.3)	5 (35.7)	0.131
Not smoking during work	236 (94.4)	103 (43.6)	133 (56.4)	
**Vaping status**				
Vaping	55 (22.0)	29 (52.7)	26 (47.3)	1.181
Not vaping	195 (78.0)	83 (42.6)	112 (57.4)	
**Vaping during work**				
Vaping during work	16 (6.4)	9 (56.3)	7 (43.8)	0.341
Not vaping during work	234 (93.6)	103 (44.0)	131 (56.0)	
**Alcohol status**				
Consumes Alcohol	36 (14.4)	25 (69.4)	11 (30.6%)	0.001
No alcohol consumption	214 (85.6)	87 (40.7)	127 (59.3%)	
**Alcohol during work**				
Consumes alcohol during work	9 (3.6)	6 (66.7)	3 (33.3%)	0.306**
No alcohol consumed during work	241 (96.4)	106 (44.0)	135 (56.0%)	
**Monthly income** ^**a**^	2000 (1290)^#^	2000 (1400)^#^	2000 (1108)^#^	0.758*

a = Not normally distributed from normality test (Kolmogorov-Smirnov test, Skewness, Kurtosis, histogram, and Q-Q plot)

# = median (IQR)

* = Mann Whitney U test

** = Fisher Exact Test

The association between the prevalence of recent occupational industries and respondents’ characteristics and occupational factors is presented in Tables 3 and 4, respectively. Among all the respondents’ characteristics, only alcohol consumption status was associated with the occurrence of occupational injuries, whereas occupational factors such as poor knowledge (p = 0.031), poor compliance (p = 0.021), poor safety management (p = 0.021), poor safety training (p = 0.002), poor safety culture (p = 0.003), physical exposure (p < 0.001), and ergonomic exposure (p = 0.009) were significant.

**Table 4:** Association between recent occupational injuries and occupational factors among food industry workers in Selangor.

Variable	n (%)	Recent Occupational Injury		P value
		Yes	No	
		112 (44.8%)	138 (55.2%)	
**Knowledge**				
Poor knowledge	177 (70.8)	87 (49.2)	90 (50.8)	0.031
Good Knowledge	73 (29.2)	25 (34.2)	48 (65.8)	
**Compliance**				
Poor compliant	183 (73.2)	90 (49.2)	93 (50.8)	0.021
Good Compliant	67 (26.8)	22 (32.8)	45 (67.2)	
**Safety management**				
Poor management	92 (36.8)	50 (54.3)	42 (45.7)	0.021
Good management	158 (63.2)	62 (39.2)	96 (60.8)	
**Safety training**				0.002
Poor training	98 (39.2)	56 (57.1)	42 (42.9)	
Good training	152 (60.8)	56 (36.8)	96 (63.2)	
**Safety culture**				0.003
Poor culture	102 (40.8)	57 (55.9)	45 (44.1)	
Good culture	148 (59.2)	55 (37.2)	93 (62.8)	
**Physical exposure**				<0.001
Exposed	213 (85.2)	107 (50.2)	106(49.8)	
Not exposed	37 (14.8)	5 (13.5)	32(86.5)	
**Chemical exposure**				0.932
Exposed	160 (64.0)	72 (45.0)	88 (55.0)	
Not exposed	90 (36.0)	40 (44.4)	50 (55.6)	
**Ergonomic exposure**				0.009
Exposed	198 (79.2)	97(49)	101 (51)	
Not exposed	52 (20.8)	15 (28.8)	37 (71.2)	
**Rest at work**				0.078
Inadequate rest	73 (29.2)	39 (53.4)	34 (46.6)	
Adequate rest	177 (70.8)	73 (41.2)	104 (58.8)	
**Working status**				0.366
Permanent worker	185 (74.0)	86 (46.5)	86 (53.5)	
Temporary worker	65 (26.0)	26 (40.0)	39 (60.0)	
**Working hours**				0.319
Long working hours	159 (63.6)	75 (47.2)	84 (52.8)	
Normal working hour	91 (36.4)	37 (40.7)	54 (59.3)	
**Working years** ^**a**^	2 (5)^#^	2 (5)^#^	2 (5)^#^	0.759*

a = Not normally distributed from normality test (Kolmogorov-Smirnov test, Skewness, Kurtosis, histogram, and Q-Q plot)

* = Mann Whitney U test

# = median (IQR)

Table 5 shows the SLR analyses, indicating significant associations between the prevalence of recent occupational injuries and alcohol consumption (cOR = 3.32, 95% CI = 1.101, 3.558, p = 0.022), poor safety management (cOR = 1.84, 95% CI = 1.096, 3.100, p = 0.021), poor safety training (cOR = 2.29, 95% CI = 1.361, 3.838, p = 0.002), poor safety culture (cOR = 2.14, 95% CI 1.281, 3.580, p = 0.004), physical exposure (cOR = 6.46, 95% CI = 2.425, 17.214, p < 0.001), and ergonomic exposure (cOR = 2.37, 95% CI = 1.223, 4.590, p = 0.011). In the MLR model (Table 6), the predictors for recent occupational injuries among food industry workers were Malay (aOR = 2.60, 95% CI = 1.116, 6.035, p = 0.027), alcohol consumption (aOR = 5.31, 95% CI = 2.042,13.779, p = 0.001), poor knowledge (aOR = 1.98, 95% CI = 1.059, 3.691, p = 0.032), poor safety culture (aOR = 2.44, 95% CI = 1.372, 4.342, p = 0.002), and exposure to physical hazard (aOR = 8.88, 95% CI = 3.031, 26.014, p < 0.001).

**Table 5:** Simple Logistic Regression (SLR) analysis between recent occupational injuries and potential factors among food industry workers in Selangor.

	Simple Logistic Regression				
Factors	Unadjusted Coefficient	S.E.	Crude OR	95% CI for (lower, upper)	P value
**Age**	0.006	0.013	1.006	0.980, 1.033	0.671
**Gender**					
Female	0.070	0.257	1.07	0.648, 1.775	0.748
Male			1.00		
**Ethnicity**					
Malay	0.215	0.336	1.24	0.642, 2.397	0.522
Chinese	-0.210	0.370	0.81	0.393, 1.673	0.569
Indian	0.472	0.469	1.60	0.639, 4.023	0.314
Others					
**Nationality**					
Local	0.461	0.458	1.585	0.647, 3.887	0.314
Foreigner			1.00		
**Educational level**					
Primary	0.920	.497	2.51	0.947, 6.645	0.064
Secondary	0.910	.519	2.49	0.899, 6.867	0.079
Diploma	1.253	.779	3.50	0.760, 16.118	0.108
Bachelor	1.609	.956	5.00	0.767, 32.574	0.092
Master	1.504	.759	4.50	1.017, 19.902	0.047
Others			1.00		
**Smoking status**					
Smoking	0.308	0.272	1.36	0.798,2.321	0.257
Not smoking			1.00		
**Smoking during work status**					
Smoking during work	0.843	0.573	2.32	0.756, 7.145	0.141
Not smoking during work			1.00		
**Vaping status**					
Vaping	0.409	0.306	1.51	0.825, 2.744	0.182
Not vaping			1.00		
**Vaping during work status**					
Vaping during work	0.492	0.521	1.64	0.589, 4.539	0.345
Not vaping during work			1.00		
**Alcohol status**					
Consumes alcohol	1.199	0.388	3.32	1.552, 7.093	0.002
No alcohol consumption			1.00		
**Alcohol during work status**					
Consumes alcohol during work	0.935	0.719	2.55	0.622, 10.423	0.193
No alcohol consumed during work			1.00		
**Monthly income**	0.000	0.000	1.000	1.000,1.000	0.263
**Knowledge**					
Poor knowledge	0.618	0.289	1.86	1.054, 3.269	0.032
Good Knowledge			1.00		
**Compliance**					
Poor compliant	0.683	0.299	1.98	1.101, 3.558	0.022
Good Compliant			1.00		
**Safety management**					
Poor management	0.612	0.265	1.84	1.096, 3.100	0.021
Good management			1.00		
**Safety training**					
Poor training	0.827	0.264	2.29	1.361, 3.838	0.002
Good training			1.00		
**Safety culture**					
Poor culture	0.762	0.262	2.14	1.281, 3.580	0.004
Good culture			1.00		
**Physical exposure**					
Exposed	1.866	0.500	6.46	2.425, 17.214	<0.001
Not exposed			1.00		
**Chemical exposure**					
Exposed	0.022	0.265	1.02	0.608, 1.719	0.932
Not exposed			1.00		
**Ergonomic exposure**					
Exposed	0.862	0.337	2.37	1.223, 4.590	0.011
Not exposed			1.00		
**Rest at work**					
Inadequate rest	0.491	0.280	1.63	0.944, 2.829	0.079
Adequate rest			1.00		
**Working status**					
Temporary worker	0.265	0.293	1.30	0.734, 2.314	0.366
Permanent worker			1.00		
**Working hours**					
Long working hours	0.265	0.266	1.30	0.774, 2.195	0.320
Normal working hour			1.00		
**Working years**	-0.022	0.020	0.98	0.940, 1.019	0.290

S.E = Standard Error, OR = Odd Ratio, CI = Confidence interval

**Table 6:** Multiple Logistic Regression (MLR) analysis between recent occupational injuries and the predictors among food industry workers in Selangor.

	Multiple Logistic Regression				
Factors	Adjusted Coefficient	S.E.	Adjusted OR	95% CI for (lower, upper)	P value
**Ethnicity**					
Malay	0.954	0.431	2.60	1.116, 6.035	0.027
Chinese	-0.247	0.422	0.78	0.342, 1.786	0.558
Indian	0.867	0.540	2.38	0.826, 6.862	0.108
Others			1.00		
**Alcohol status**					
Consumes alcohol	1.669	0.487	5.31	2.042,13.779	0.001
No alcohol consumption			1.00		
**Knowledge**					
Poor knowledge	0.682	0.319	1.98	1.059, 3.691	0.032
Good Knowledge			1.00		
**Safety culture**					
Poor culture	0.892	0.294	2.44	1.372, 4.342	0.002
Good culture			1.00		
**Physical exposure**					
Exposed	2.184	0.548	8.88	3.031, 26.014	<0.001
Not exposed			1.00		
**Constant**	-2.412				

S.E. = Standard Error, OR = Odd Ratio, CI = Confidence interval

Multiple Logistic Regression (backward LR and forward LR)

No interaction (p > 0.05), no multicollinearity (VIF < 10), no influential outlier

Hosmer-Lemeshow goodness-of-fit test p = 0.944 (model fits)

Omnibus Model of Coefficients p < 0.001, Cox & Snell R^2^ = 0.186, Nagelkerke R^2^ = 0.249

Overall accuracy = 66.8%, Sensitivity = 75.4%, Specificity = 56.3%.

## Discussion

This study assessed the prevalence and predictors of recent occupational injuries among food industry workers in Selangor, Malaysia. A relatively moderate response rate (73.0%) was achieved for the data collection given that most of the respondents completed the survey during their peak working hours and busy schedules.

Descriptively, most of the food industry workers in this study were young adults (median age of 26), Malays (56.4%), Malaysian citizens (90.8%), and had attended a secondary school (49.6%) (Table 2). Young adults are more likely to constitute the larger proportion of food industry workers since the service requires physical activities, long working hours and tasks that may not be effectively executed by teenagers and aged individuals. It is also not surprising that Malays and Malaysians were predominant since this study was conducted in various food service outlets in Selangor. The demand for low to medium-skilled workers in the food service outlets may also explain the higher proportion of respondents with only secondary school qualifications.

The prevalence of recent injuries among food industry workers was 44.8% (Table 1), which is lower than the overall prevalence (73.5%) of work-related injuries among workers of industry in Malaysia [20] but similar to a study conducted in Greece with 44.3% of recent injuries among restaurant workers [15]. The substantial gap in findings might be explained by the fact that the research population was different. This study only included workers in the food industry, whereas the study among workers of industry in Malaysia [20] included workers from other high-risk industries and a broader research population from other states in Malaysia. Thus, the present findings should only be used to refer to food industry workers for an accurate representation. As shown in Table 2, the most common types of injuries reported were cuts, falls, burns, lower back pain, and sprains, which aligned with data from the Department of Statistics Malaysia [6]. Cuts and burns may arise from the handling of cooking-related utensils and exposure to fire accidents during food preparation [15]. Lower back pain and sprains, on the other hand, may stem from prolonged standing time and awkward postures portrayed by food industry workers during long working hours.

Several factors were identified as predictors of recent occupational injuries among the surveyed food industry workers. Specifically, this study found that alcohol consumption increases the risk of experiencing recent occupational injury up to 5.3 times compared to those who do not consume alcohol (Table 6). This finding is supported by previous studies. A study on alcohol consumption and related harms reported that over 40% of Australian workers consumed alcohol at risk or high-risk levels of injuries [21]. Similarly, data from four national alcohol surveys between 2000 and 2015 found that the risk of injury would increase at relatively low levels of alcohol consumption [22]. Alcohol-attributable injury accounted for approximately one-tenth of the total impact of alcohol on health at 9.9% and 12.6% in low- and high-income countries, respectively [23, 24]. This phenomenon can be explained by levels of alcohol consumption that can impair alertness, motor skills, coordination, cognitive function, and reaction time. These impairments can lead to errors, risk-taking behaviour, impaired communication, and teamwork breakdown [25–27], thereby contributing to accidents and injuries among workers.

Worker knowledge was identified as a significant factor associated with recent occupational injuries among food industry workers in Selangor. Respondents with poor knowledge demonstrated 2 times higher odds of experiencing recent occupational injury compared to those with good knowledge (Table 6). This finding aligns with a previous study that revealed a strong correlation between worker knowledge and occupational injury prevention [28]. This event can be explained as workers with higher levels of occupational safety and health (OSH) knowledge displayed better hazard recognition skills, practised greater safety compliance, and experienced fewer workplace injuries. When workers possess knowledge about workplace hazards, they are more capable of identifying risks and taking necessary precautions to prevent accidents. Adequate knowledge enables workers to follow safety procedures, utilise personal protective equipment (PPE), and actively participate in risk assessment processes [29].

Poor safety culture was identified as a predictor of recent occupational injuries among food industry workers. This study found that a poor safety culture can increase the risk of workers experiencing recent occupational injuries up to 2.4 times compared to those that have a good safety culture. Similar results were reported in a study among manufacturing companies in which employers’ lack of knowledge about safety issues, employees’ lack of cooperation and engagement in safety, and a lack of belief in the use of PPE were the contributing factors to injuries [30]. Another study involving small and medium-scale flour mill workers emphasised the significance of a weak safety culture and reporting mechanisms in relation to workplace injuries [31]. The odds of accidents and injuries can be increased by inadequate safety communication channels and a disregard for safety precautions due to a lack of emphasis on safety in the corporate culture. Establishing an efficient safety reporting process and promoting a strong safety culture that values preventative safety measures are crucial for preventing workplace injuries [31–33].

In this study, food industry workers who were exposed to physical hazards faced 8.9 times higher risks of experiencing recent occupational injuries compared to those who were not exposed to physical hazards (Table 6). This finding is consistent with a previous study conducted among commercial restaurant kitchens that demonstrated a significant association between physical exposure and occupational injuries among food industry workers [34]. Their study revealed that kitchen duties that require strenuous physical labour or prolonged periods of uncomfortable posture might lead to slip-and-fall incidents or back injuries among workers. A similar scenario was observed in the present study as falls, slips and cuts accounted for the majority of types of recent occupational injuries reported by the respondents. These findings underscored the importance of addressing physical exposure as a key factor in promoting worker safety and preventing injuries in this industry [34].

This study also found that Malay workers were significantly associated with recent occupational injuries. The Malay food industry workers were 2.6 times at more risk of experiencing recent occupational injuries relative to workers of other ethnicities (Table 6). The importance of ethnic diversity and socio-economic status among different races have been highlighted in a few previous studies as risk factors for occupational injuries [35, 36]. A study on racial and ethnic differences in the frequency of workplace injuries suggested that disparities in economic opportunities can expose groups of minorities to greater risks of workplace injury and disability [35]. Similarly, occupational injuries among workers from different ethnicities concluded that the high-risk nature of certain occupations and the presence of ethnic minorities in these high-risk occupations have contributed to the increased rate of fatal occupational injuries. Thus, unskilled occupations warrant effective safety training programmes and enforcement of laws to ensure safer workplaces [36]. Nevertheless, this finding should be interpreted with caution given that the majority of food industry workers recruited in the present study were Malays.

Further analysis of the safety culture component in this study shows a significant agreement among respondents, with 37.2% agreeing and 42.0% strongly agreeing that all workers in the food industry should receive safety training. This agreement highlights the industry’s joint aim for improved safety measures and training, highlighting the importance of proactive safety measures in reducing the risk of workplace injuries. Furthermore, a substantial percentage of participants, with 23.6% agreeing and 24.0% strongly agreeing, are willing to bear the financial burden for safety training if such programs were available. This willingness signifies a commendable commitment to personal and workplace safety and bolsters the argument for the urgent need to make safety training more accessible, easing the financial burden on individual workers. These findings collectively form a compelling case for policymakers and stakeholders to develop intervention programs that meet the expressed demands of food industry workers, ultimately enhancing safety and well-being within the food industry.

### Limitations and strengths

There are several limitations to be considered when interpreting the findings from this study. The absence of incentives for the respondents, constraints imposed by peak work hours, limitations to specific geographical areas, and potential biases associated with the research design have all contributed to the limitations of this study. These factors may affect the generalisability, validity, and reliability of the results. Future research with larger and more diverse samples, offering incentives for respondents, and considering off-peak data collection hours are needed to strengthen the findings. Addressing these issues will enhance the generalisability and applicability of the results to the broader population.

Despite the aforementioned limitations, this study has several notable strengths. The comprehensive approach to investigating various factors associated with occupational injuries, including both individual and occupational factors, has provided a more holistic understanding of the subject. By examining multiple dimensions, this study can contribute to a better grasp of the relationships between the factors and predictors of recent occupational injuries among food industry workers. Additionally, the direct and personal approach to contacting respondents facilitated easier access, improved engagement, and led to potentially higher response rates. This approach enhanced the quality of data collection and increased the validity and dependability of the findings. Furthermore, the use of a brief and simple questionnaire minimised participants’ cognitive load and allowed for precise and understandable responses, which made this study more accessible and applicable to a wider audience.

### Recommendations

The findings can provide valuable insights into the predictors of recent occupational injuries among food industry workers, but further research is necessary to validate and expand upon these results. By addressing the limitations and building upon the strengths, future studies can elucidate occupational injuries and support the development of effective interventions to improve workplace safety and protect the well-being of food and beverage workers. This study strongly recommends the collaboration between the Department of Occupational Safety and Health (DOSH), the National Institute of Occupational Safety and Health (NIOSH), and the Social Security Organization (SOCSO) to develop comprehensive and targeted interventions aimed at addressing the specific predictors identified in this research.

First, the organisations highlighted above need to address the issue of alcohol consumption effectively. Implementing stringent policies and awareness campaigns that discourage alcohol consumption can significantly reduce the likelihood of workplace incidents and contribute towards a safer working environment. Second, emphasis should be placed on improving workers’ knowledge and compliance with safety protocols. Collaborative efforts should be made to organise regular training sessions and workshops conducted by NIOSH to educate workers about potential hazards and instil a culture of safety consciousness. By enhancing workers’ understanding of safety practices, employers can foster a workforce that is more proactive in preventing accidents and injuries. Third, creating a positive work safety culture is vital in cultivating an environment where safety is prioritised. DOSH, NIOSH, and SOCSO should jointly launch awareness campaigns that encourage open communication, reporting of safety concerns, and the recognition of safe practices. A culture that values safety will lead to increased compliance with safety regulations and promote a collective responsibility for maintaining a secure workplace.

Furthermore, the relevant organisations need to focus on implementing effective control measures for physical exposure hazards, with particular attention to the needs of Malay workers. By developing industry-specific guidelines and standards, DOSH, NIOSH, and SOCSO can cater to the unique risks faced by different sectors in ensuring that safety measures are tailored to the specific working conditions of workers. By collectively prioritising these recommended factors and drawing on the expertise of each organisation, DOSH, NIOSH, and SOCSO can synergise their efforts in creating safer work environments across various industries in Malaysia. This collaborative approach will not only minimise the risk of occupational injuries but also demonstrate a proactive commitment towards ensuring the well-being and safety of the nation’s workforce.

## Conclusion

This study revealed a high prevalence of occupational injuries among food industry workers. The findings highlighted the importance of addressing alcohol consumption, improving worker knowledge, enhancing work safety culture, and better control measures on physical exposure hazards, especially among Malay food industry workers. By prioritising these factors, employers can create safer work environments and minimise the risk of occupational injuries.

### Data management

Online questionnaires were used for pseudonymised data collection. Study data and personal information were stored using Microsoft Excel. These data were transferred to the records and documentation system under Universiti Putra Malaysia. The data will be destroyed five years later. Additionally, any study respondents who have requested to be briefed about the findings will be informed via an email correspondence

## References

[1] Laws of Malaysia Act 281 Food Act. (2006). Laws of Malaysia Act 281 Food Act 1983. 1–24. http://www.vertic.org/media/National%20Legislation/Malaysia/MY_Food_Act.pdf

[2] Md Noor NRA, Zaman LK, Yaacob N, & Hassan MS. A conceptual study on the awareness of foodborne disease and food handling regulation among food handlers. E-proceeding 8th International Conference on Public Policy And Social Science (ICoPS) 2021. https://ir.uitm.edu.my/id/eprint/54090/1/54090.pdf

[3] Saad M, Toh PS., & Mohamed Adil MA. Hygiene Practices of Food Handlers at Malaysian Government Institutions Training Centers. Social and Behavioural Sciences 85, 2013,118–127. 10.1016/j.sbspro.2013.08.344

[4] Europe F. D. (2018). Data & trends EU food and drink industry 2017. Food Drink Eur, 1–25. https://www.fooddrinkeurope.eu/resource/data-trends-of-the-european-food-and-drink-industry-2018-2/

[5] Musungwa T, & Kowe P. Effects of occupational health and safety management systems implementation in accident prevention at a Harare beverage company, Cogent Engineering, 2022, 9:1, 2124638, 10.1080/23311916.2022.2124638.

[6] Department of Statistics Malaysia: Press release annual economic statistics 2018 Food and Beverage services. Department of Statistics Malaysia, March, 1–2. https://www.dosm.gov.my/portal-main/release-archive/04f08c71-b765-11ed-9995-1866daa77ef9

[7] Bonsu WS, Adei D, & Agyemang-Duah W. Exposure to occupational hazards among bakers and their coping mechanisms in Ghana Exposure to occupational hazards among bakers and their coping mechanisms in Ghana. Cogent Medicine. 2020, 7. 1–20. 10.1080/2331205X.2020.1825172.

[8] Ngajilo D, & Jeebhay MF. Occupational injuries and diseases in aquaculture–A review of literature, Aquaculture, 2019, 507, 40–55. 10.1016/j.aquaculture.2019.03.053.

[9] Md Zamri ASS, Saruddin MZ, Harun A, Abd. Aziz SF, Aizad Za’bah AK, Dapari R, et al. (2023) Factors associated with occupational asthma among food industry workers: A systematic review. PLoS ONE 18(6): e0287040. doi: 10.1371/journal.pone.0287040 37307252 PMC10259786

[10] Lippert J, Rosing H, Tendick-Matesanz F. The health of restaurant work: A historical and social context to the occupational health of food service. Am J Ind Med, 2020 63(7), 563–576. doi: 10.1002/ajim.23112 32329097

[11] Suzman MS, Sobocinski K, Himel H, Yurt RW. Major burn injuries among restaurant workers in New York City: an underappreciated public health hazard. J Burn Care Rehabil, 2001 22(6), 429–434. doi: 10.1097/00004630-200111000-00014 . 11761396

[12] International Labour Organization (ILOSTAT). Occupational Safety and Health Statistics (OSH database). https://ilostat.ilo.org/resources/concepts-and-definitions/description-occupational-safety-and-health-statistics/ [Accessed 25th July 2023]

[13] Laws of Malaysia, Act 514. Occupational Safety and Health, Act 1994. https://www.dosh.gov.my/index.php/legislation/acts-legislation/23-02-occupational-safety-and-health-act-1994-act-514/file

[14] DOL U.S., BLS (2020). "Table 2 ‐ Number of cases ‐ detailed industry level. Summary tables. 2022. Washington, DC: U.S. DOL, BLS, 2020. Apr. 3, 2022. https://www.bls.gov/iif/oshwc/osh/os/summ2_00_2020.xlsx

[15] Vilelmine C. Covariates of occupational accident occurrence in the restaurant sector in Greece: the case of the restaurants in the piraeus municipality. Health Science Journal. 2011; 5(3): 196–203. https://www.hsj.gr/abstract/covariates-of-occupational-accident-occurrence-in-the-restaurant-sector-in-greece-the-case-of-the-restaurants-in-the-piraeus-municipality-3380.html

[16] DOSH. (2021). Occupational Poisoning and Diseases Statistics 2019. https://www.dosh.gov.my/index.php/ms/competent-person-form/occupational-health/statistics/occupational-diseases-and-poisoning-statistic/3869-2019

[17] Hoffmann J. The Top 5 Workplace Injuries Among Food Industry Workers. St Louis Workers Compensation Attorney & Work Injury Lawyer. 2022. https://www.hoffmannworkcomp.com/the-top-5-workplace-injuries-among-food-industry-workers/

[18] DOSM. (2019). Department of Statistics Malaysia: Pocket Stats Q3 2022. www.dosm.gov.my

[19] Wang H., & Chow S. C. (2007). Sample Size Calculation for Comparing Proportions. Wiley Encyclopedia of Clinical Trials. 10.1002/9780471462422.eoct005

[20] Obi A.N., Ariffin A.A., & Zainuddin H. Factors Associated with Work-Related Injuries among Workers of an Industry in Malaysia. International Journal of Public Health and Clinical Sciences. 2017 4. 97–108. https://www.researchgate.net/publication/329206251_Factors_Associated_with_Work_Related_Injuries_among_Workers_of_an_Industry_in_Malaysia#fullTextFileContent

[21] Roche A, Kostadinov V, Fischer J, Nicholas R, O’Rourke K, Pidd K et al. Addressing inequities in alcohol consumption and related harms. Health Promot Int. Suppl. 2015; 20–35. doi: 10.1093/heapro/dav030 . 26420810

[22] Cherpitel CJ, Ye Y, Kerr WC. Risk of Past Year Injury Related to Hours of Exposure to an Elevated Blood Alcohol Concentration and Average Monthly Alcohol Volume: Data from 4 National Alcohol Surveys (2000 to 2015). Alcohol Clin Exp Res. 2018 Feb;42(2):360–368. doi: 10.1111/acer.13561 Epub 2017 Dec 19. ; PMCID: PMC5785417. 29160960 PMC5785417

[23] Institute for Health Metrics and Evaluation. GBD Results Tool: Global Health Data Exchange. University of Washington; Seattle, WA, USA: 2021. https://ghdx.healthdata.org

[24] Chikritzhs T., & Livingston M. (2021). Alcohol and the Risk of Injury. Nutrients, 13(8). doi: 10.3390/nu13082777 34444939 PMC8401155

[25] Brumback T, Cao D, King A. Effects of alcohol on psychomotor performance and perceived impairment in heavy binge social drinkers. Drug Alcohol Depend. 2007 Nov 2;91(1):10–7. doi: 10.1016/j.drugalcdep.2007.04.013. Epub 2007 Jun 8. ; PMCID: PMC2764986. 17560739 PMC2764986

[26] Garrisson Harriet, Scholey Andrew, Ogden Edward, Benson Sarah. The effects of alcohol intoxication on cognitive functions critical for driving: A systematic review. Accident Analysis & Prevention, 2021, 154, 106052, doi: 10.1016/j.aap.2021.106052 33676142

[27] Cheng CH, Huang LL, Kao WT, Su CY, Chou FH, Hsieh KY. Cognitive function and alcohol use disorder: Path analysis for a cross-sectional study in Taiwan. Taiwan J Psychiatry 2021;35:124–31. doi: 10.4103/TPSY.TPSY_25_21

[28] Kao Kuo-Yang, Spitzmueller Christiane, Cigularov Konstantin & Thomas Candice L. (2019) Linking safety knowledge to safety behaviours: a moderated mediation of supervisor and worker safety attitudes, European Journal of Work and Organizational Psychology, 28:2, 206–220, doi: 10.1080/1359432X.2019.1567492

[29] Clarke S., & Robertson I. T. The Relationship Between Safety Knowledge, Safety Motivation, and Safety Performance: A Meta-Analytic Review. Journal of Occupational Health Psychology. 2005; 10(4): 315–327. 10.1037/1076-8998.10.4.315 17059296

[30] Ghahramani A., & Amirbahmani A. A qualitative investigation to discover causes of occupational injuries and preventive countermeasures in manufacturing companies. Heliyon. 2022; 8(9), e10501. doi: 10.1016/j.heliyon.2022.e10501 36097477 PMC9463575

[31] Mekonnen T. H., Dessie A., & Tesfaye A. H. Respiratory symptoms related to flour dust exposure are significantly high among small and medium scale flour mill workers in Ethiopia: a comparative cross-sectional survey. Environmental Health and Preventive Medicine.2021; 26(1). 10.1186/s12199-021-01019-y PMC8479999 34587904

[32] Clarke S., & Ward K. Effect of Safety Compliance on Workplace Injury Rates in Manufacturing Settings. Journal of Occupational and Organisational Psychology. 2006; 79(4); 473–482. 10.1348/096317905X53267

[33] Kim Yangho, Park Jungsun, Park Mijin. Creating a Culture of Prevention in Occupational Safety and Health Practice. Safety and Health at Work, 2016, 7,2, 89–96, doi: 10.1016/j.shaw.2016.02.002 27340594 PMC4909854

[34] Byung Y. J. Cooking processes and occupational accidents in commercial restaurant kitchens. Safety Science. 2015; 80: 87–93. 10.1016/j.ssci.2015.07.014

[35] Seabury SA, Terp S & Boden LI. Racial and Ethnic Differences in the Frequency of Workplace Injuries and the Prevalence of Work-Related Disability. Health Aff (Millwood). 2017 Feb 1; 36(2): 266–273. doi: 10.1377/hlthaff.2016.1185 28167715 PMC6198680

[36] Mekkodathil A, El-Menyar A, & Al-Thani H. Occupational injuries in workers from different ethnicities. Int J Crit Illn Inj Sci. 2016 Jan-Mar; 6(1): 25–32. doi: 10.4103/2229-5151.177365 27051619 PMC4795358

